# Early Performance of a Collared, Triple-Tapered Cementless Femoral Stem in Primary Total Hip Arthroplasty

**DOI:** 10.3390/jcm15166343

**Published:** 2026-08-17

**Authors:** Thomas L. Bradbury, Charlotte C. Baker, Zachary M. Ricciardelli, Evan Bilson, Joseph M. Schwab, George N. Guild

**Affiliations:** Total Joint Specialists, Northside Hospital Forsyth, Cumming, GA 30041, USAzach.ricciardelli@gmail.com (Z.M.R.); joeschwabmd@gmail.com (J.M.S.);

**Keywords:** triple-taper stem, automated impactor, early complications

## Abstract

**Background/Objectives**: Collared triple-tapered cementless femoral stems are designed to optimize metaphyseal fit, stability, and osseointegration following total hip arthroplasty (THA). This study evaluated 90-day revision and complication rates, short-term functional recovery, and operative characteristics of a modern collared triple-tapered stem in a same-day discharge ambulatory surgery center cohort. **Methods**: This retrospective cohort study reviewed 525 consecutive primary direct anterior THAs performed by 15 fellowship-trained arthroplasty surgeons at one group operating across several ASCs between September 2024 and August 2025. Ninety-day all-cause revision, stem-specific revision, periprosthetic fracture, and other early postoperative complications were evaluated. Kaplan–Meier estimates were used to account for variable follow-up within the 90 days. PROMs (HOOS-JR, FJS, VR-12) were collected preoperatively and at 12 weeks. Operative time was evaluated using within-surgeon analyses restricted to surgeons who used both an automated impaction device (AID) and manual impaction. **Results**: At a mean effective follow-up of 110 ± 39 days, the 90-day Kaplan–Meier revision-free estimate was 99.62% for all-cause revision and 99.81% for stem revision. Two patients (0.4%) underwent revision (one Vancouver B2 fracture; one infection treated with DAIR with stem retention). The early periprosthetic fracture rate was 0.2%. Five patients (1.0%) experienced dislocation, all of whom were managed with closed reduction. HOOS-JR improved by 28.7 points and FJS by 41.9 points at 12 weeks (both *p* < 0.0001), with 84.2% and 85.8% MCID achievement, respectively. Seven surgeons used both techniques, contributing 384 AID and 58 manual cases to the within-surgeon analysis. AID use was not associated with operative time after accounting for surgeon (adjusted difference, 0.18 min; 95% CI, −2.04 to 2.39; *p* = 0.8764). **Conclusions**: This collared, triple-tapered cementless stem was associated with low 90-day revision and complication rates and clinically meaningful short-term functional improvement in a same-day discharge ambulatory population. AID use was not associated with operative time after accounting for surgeon, although the study was not powered to compare uncommon complications between impaction methods.

## 1. Introduction

Total hip arthroplasty (THA) is one of the most successful and frequently performed orthopedic procedures, demonstrating survivorship rates of 93–100% at 20-year follow-up [1,2]. With the adoption of highly cross-linked polyethylene acetabular inserts, the risk of long-term wear-related failure after THA has been dramatically reduced [3,4,5].

Cementless femoral components are preferred in the majority of primary THA cases in the United States, with modern design innovations aimed at optimizing initial fit and stability to facilitate osseointegration [6,7]. Despite the promising long-term performance data of cementless femoral component fixation, early mechanical complications, including periprosthetic fracture and aseptic failure of osseointegration, remain clinically meaningful causes of early revision and patient morbidity [8,9,10,11]. In ambulatory and same-day-discharge (SDD) settings, these early events carry disproportionate clinical, operational, and patient-experience consequences, making early stability and reproducible fixation critical implant attributes. These early events may reflect implant–bone mismatch, inadequate initial stability, or other patient- and procedure-related factors.

Common cementless femoral stem designs include tapered-wedge, fit-and-fill, and triple-tapered geometries, each relying on different patterns of metaphyseal and/or diaphyseal fixation. Triple-tapered stems are designed to achieve initial axial and rotational stability through metaphyseal fixation [12,13]. The stem evaluated in the present study is a collared, triple-tapered titanium alloy design with a proximal titanium and hydroxyapatite coating intended to support biologic fixation [14,15]. Multiple neck-offset configurations are available to accommodate anatomical variations [16,17].

Automated impaction systems have also been introduced as an alternative to conventional manual mallet impaction, with the aim of providing reproducible impaction while reducing physical demand on the surgeon. However, the clinical effect of automated impaction may be influenced by surgeon technique and practice patterns.

Despite favorable mid- and long-term data for several cementless stem designs, early outcome data for newer implants in same-day-discharge ambulatory pathways remain limited.

Early outcome data are essential for assessing the safety and performance of new implant designs, particularly in same-day discharge settings where patient selection, perioperative protocols, and recovery expectations differ from inpatient environments. The purpose of this retrospective review was to characterize 90-day revision and complication rates, including intraoperative and postoperative periprosthetic fractures, dislocations, infections, reoperation, and revision for any cause, and to evaluate short-term patient-reported outcomes following primary THA with this collared triple-tapered stem in a same-day discharge cohort.

## 2. Materials and Methods

After the study was determined to be exempt by the Northside Hospital Institutional Review Board, we retrospectively reviewed all patients who underwent primary THA with a Zimmer Z1 femoral stem (Zimmer Biomet, Warsaw, IN, USA) from September 2024 to August 2025, by fifteen fellowship-trained arthroplasty surgeons in one group operating across several ambulatory surgery centers (ASCs). Patients were included if they were aged 18 years or older and had at least 90 days of clinical follow-up, completed 12-week PROMs, or experienced a complication within 90 days of surgery. The surgical technique was similar for all surgeons, utilizing a direct anterior approach. The HAMMR™ Automated Hip Impaction System (Zimmer Biomet, Warsaw, IN, USA), a powered automated impaction device (AID), was used according to individual surgeon preference and practice pattern; no standardized patient-selection algorithm determined AID use. In cases where the AID was not used, conventional manual mallet impaction was performed.

We reviewed operative notes and clinical visit notes from the preoperative appointment until the most recent follow-up appointment. Patient demographic data (age, sex, body mass index (BMI), age-adjusted Charlson Comorbidity Index (CCI)), surgical variables, including implant sizes, AID use, intraoperative complications, operative time (start of incision to start of closure), and 90-day events, including revision, reoperation, readmission, ED visit, dislocation, fracture, and infection, were recorded. Operative time was defined as the interval from incision to the start of closure. Because AID use varied substantially by surgeon, the primary operative-time analysis was restricted to surgeons who used both AID and manual impaction. Linear regression models with surgeon fixed effects were used to estimate the within-surgeon association between AID use and operative time. A second model was additionally adjusted for age, sex, body mass index, Charlson Comorbidity Index, and femoral stem size.

The prespecified 90-day operative endpoints were reoperation, all-cause revision, and stem revision. Reoperation was defined as any return to the operating room related to the index THA. All-cause revision was defined as exchange, removal, or addition of any prosthetic component, including modular head or liner exchange. Stem revision was defined as the removal or exchange of the femoral stem. Accordingly, debridement, antibiotics, and implant retention with modular component exchange was classified as an all-cause revision but not a stem revision, whereas cable-only reoperation without component exchange was classified as a reoperation but not a revision. Additional 90-day outcomes included periprosthetic fractures, infections, dislocations, emergency department visits, and hospital readmissions. Periprosthetic joint infection was defined as a clinically diagnosed postoperative infection requiring operative treatment. Periprosthetic femoral fracture was defined as a postoperative femoral fracture confirmed on imaging and documented by the treating surgeon. Aseptic loosening was recorded only when clinically diagnosed as the indication for revision; no standardized radiographic assessment for loosening was performed.

Secondary outcomes included patient-reported outcome measures (PROMs). The PROMs examined included the Veterans RAND 12-Item Health Survey (VR-12; physical and mental components), Hip Disability and Osteoarthritis Outcome Score for Joint Replacement (HOOS-JR), and the Forgotten Joint Score (FJS) [18,19,20]. PROMs were collected preoperatively and at 12 weeks postoperatively. Procedure satisfaction was assessed at 12 weeks using a 5-point Likert scale ranging from very dissatisfied (1) to very satisfied (5).

Distributional assumptions for paired PROM change scores were assessed using the Shapiro–Wilk test and visual inspection of Q–Q plots. VR-12 MCS, VR-12 PCS, and FJS change scores were not normally distributed, whereas HOOS-JR change scores were. Because several PROM change distributions were non-normal and the measures are bounded clinical scales, paired preoperative and postoperative PROMs were compared consistently using the Wilcoxon signed-rank test.

Continuous variables are presented as means ± standard deviations, and categorical variables as *n* (%). For responder versus nonresponder analyses, continuous variables were compared using the Wilcoxon rank-sum test, and categorical variables were compared using the chi-square test or Fisher’s exact test when expected cell counts were small. Unadjusted operative times by impaction method were summarized descriptively. Inferential operative-time analyses were performed using linear regression with surgeon fixed effects among surgeons who used both AID and manual impaction. Effect estimates are reported as adjusted mean differences with 95% confidence intervals. A second model was additionally adjusted for prespecified case-mix variables. Complications by impaction method were reported descriptively without hypothesis testing because event counts were low. Analyses were performed in R version 4.5.1 (Vienna, Austria), with α = 0.05.

## 3. Results

### 3.1. Patient Demographics and Operative Characteristics

A total of 525 THAs in 513 patients met the inclusion criteria; 12 patients underwent staged bilateral THA during the study period. Patient demographic characteristics were summarized per unique patient, whereas surgical characteristics and clinical outcomes were analyzed per hip/procedure (Table 1). Of the 525 included hips, 522 had at least 90 days of effective follow-up. Three hips had fewer than 90 days of effective follow-up and were retained because each experienced a complication within 90 days. The cohort included 244 men (48%) and 269 women (52%) with a mean age of 66 years (range, 23–91 years). The mean body mass index was 30 kg/m^2^ (range, 19–47 kg/m^2^), and the CCI score was 2.46 ± 1.37. Osteoarthritis was the preoperative diagnosis in 509 cases (97%). The mean follow-up was 110 ± 39 days.

The mean femoral stem size was 4.85 ± 2.02 (range, 0–12). The femoral component neck configuration distribution included a standard offset in 198 hips (38%), high offset in 178 (34%), and coxa vara in 149 (28%). A 36 mm head size was most commonly used (423 hips, 81%), followed by 40 mm (55, 10%), 32 mm (43, 8.2%), and 28 mm heads (4, 0.8%). Prophylactic cables were utilized in two cases (0.4%). AID was used in 432 procedures (82%) (Table 2).

### 3.2. Ninety-Day Revision and Reoperation Rates

**Figure 1:**
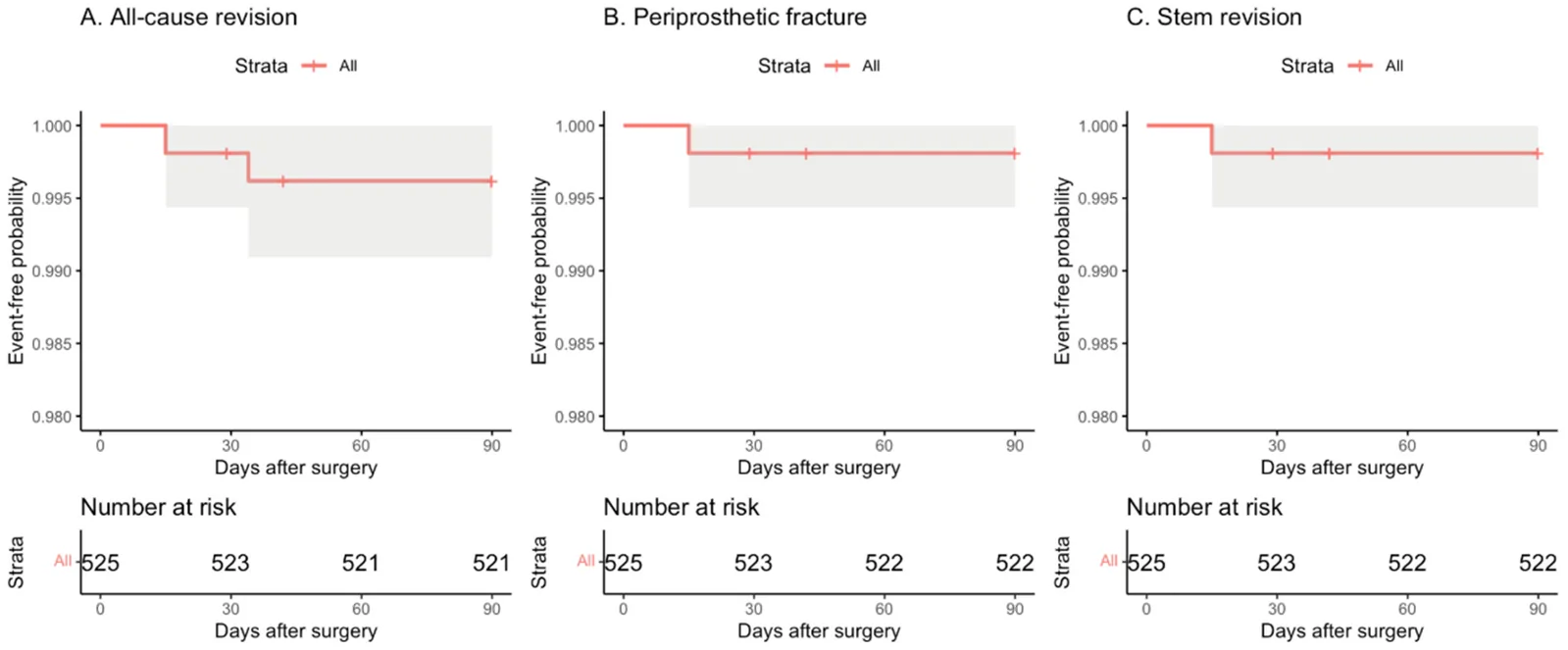
Kaplan–Meier estimates of 90-day freedom from (**A**) all-cause revision, (**C**) stem revision, and (**B**) periprosthetic fracture following primary total hip arthroplasty. Shaded areas represent 95% confidence intervals, and the number of hips at risk at 0, 30, 60, and 90 days is shown below the plot.

**Figure 2:**
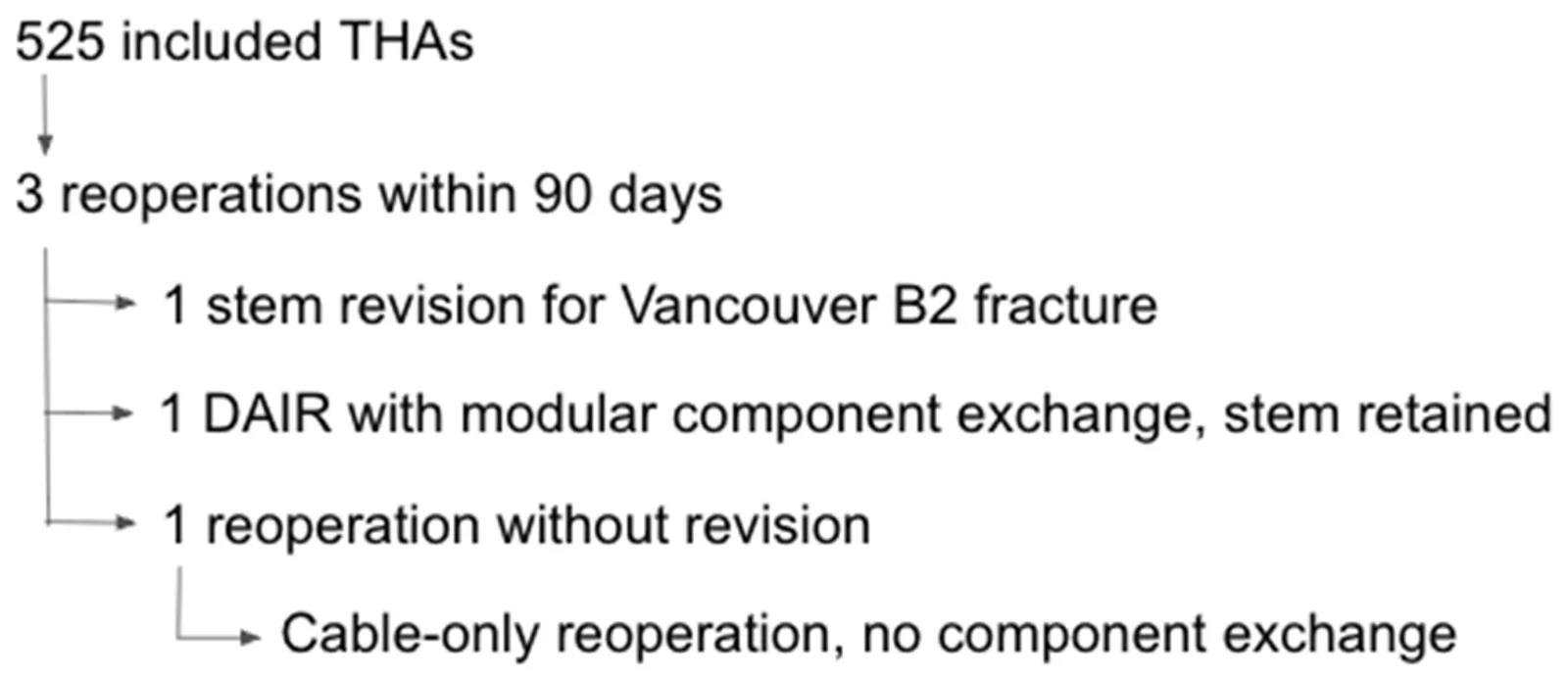
Flow diagram of 90-day reoperations and revision classification. Three hips underwent reoperation within 90 days. One Vancouver B2 periprosthetic femoral fracture required stem revision, one acute periprosthetic joint infection was treated with DAIR and modular component exchange with stem retention, and one additional hip underwent cable-only reoperation without component exchange. DAIR: debridement, antibiotics, and implant retention.

**Figure 3:**
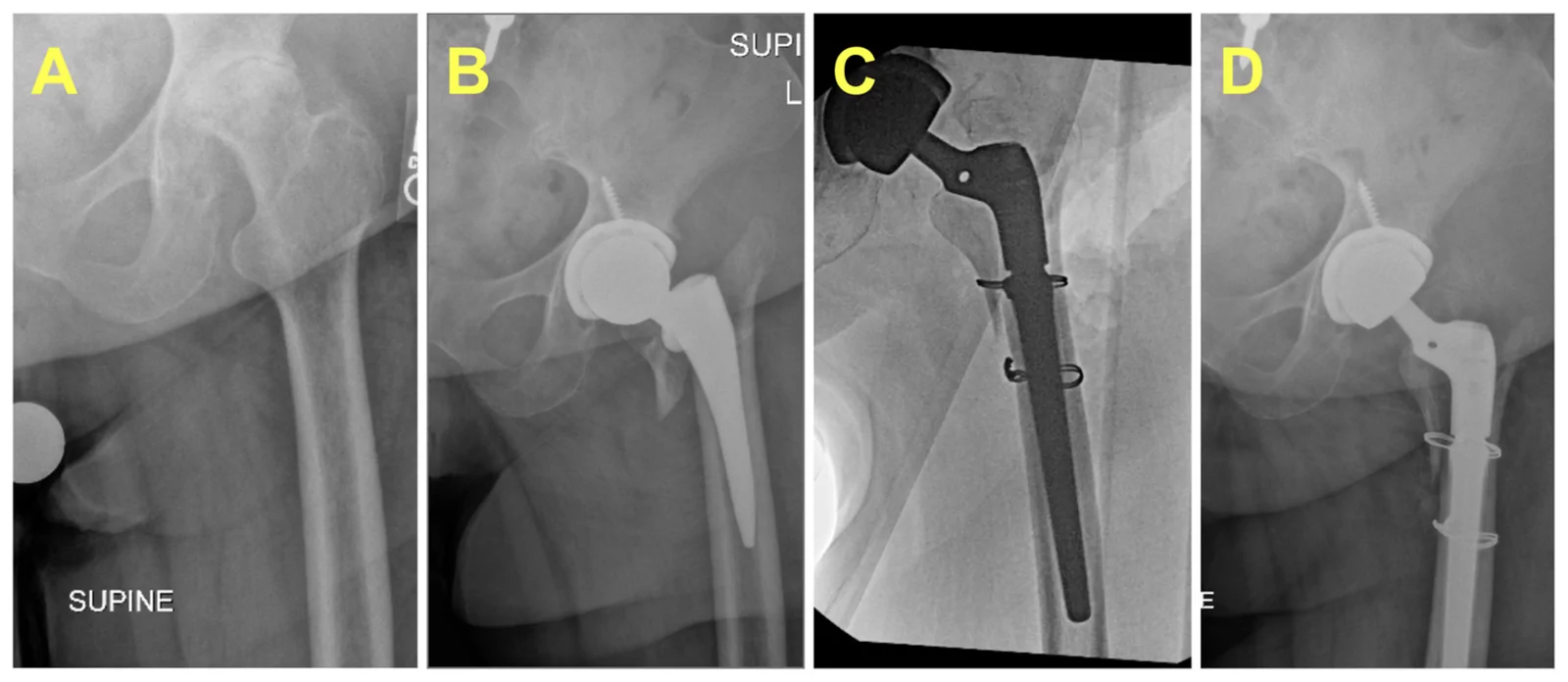
Preoperative (**A**) and postoperative (**B**–**D**) radiographs demonstrating a periprosthetic Vancouver B2 fracture. The patient began feeling pain overnight on postoperative day 15. They reported to the emergency department, and a fracture was found (**B**). The stem was revised, and two cables were placed (**C**). A 4-week postoperative radiograph following revision (**D**).

The 90-day Kaplan–Meier revision-free estimate was 99.62% for all-cause revision (95% CI, 99.09–100.00%; 521 hips at risk at 90 days) and 99.81% for stem revision (95% CI, 99.44–100.00%; 522 hips at risk at 90 days). The 90-day periprosthetic-fracture-free estimate was 99.81% (95% CI, 99.44–100.00%; 522 hips at risk at 90 days) (Figure 1). No intraoperative femoral fracture occurred. Three hips underwent reoperation within 90 days (Figure 2). Two met the definition of all-cause revision because a prosthetic component was exchanged. One Vancouver B2 periprosthetic femoral fracture was treated with stem revision and cabling and therefore counted as both an all-cause and stem revision (Figure 3). One acute periprosthetic joint infection was treated with debridement and modular component exchange with stem retention and therefore counted as an all-cause revision but not a stem revision. One additional hip underwent cable-only reoperation without component exchange and was classified as a reoperation but not a revision (Table 3). The remaining implant-related complications and medical complications are described in Table 4.

### 3.3. Postoperative Complications and Readmissions

There were four (0.8%) hospital readmissions within 90 days: one for hypoxia and cardiac issues, one for a cerebrovascular accident, one for dislocation treated with a closed reduction in the emergency department followed by admission for observation, and one for bilateral lower extremity swelling and shortness of breath. Charlson Comorbidity Index, BMI, age, and tobacco use did not differ between patients who required readmission or reoperation and those who did not.

Separately, 23 patients (4.4%) experienced adverse postoperative events that did not require reoperation or hospital readmission. This included four patients with dislocations successfully managed with a closed reduction in the emergency department without admission. The remaining 19 patients presented for other concerns, most commonly postoperative pain and falls.

### 3.4. Patient-Reported Outcomes and Satisfaction

Overall, patients reported improvement from their preoperative condition at the 12-week mark (Table 5). Paired preoperative and 12-week scores were available for 481 hips for VR-12 MCS, 481 for VR-12 PCS, 482 for HOOS-JR, and 332 for FJS. Baseline characteristics of patients with and without at least one paired PROM are shown in Supplementary Table S1. The mean improvement was 5.0 points for VR-12 MCS, 13.2 points for VR-12 PCS, 28.7 points for HOOS-JR, and 41.9 points for FJS (all *p* < 0.0001) [21,22]. Among hips with paired scores, 406 of 482 (84.2%) achieved the HOOS-JR MCID, and 285 of 332 (85.8%) achieved the FJS MCID. Satisfaction data were available for 457 hips, of whom 438 (95.8%) reported being satisfied or very satisfied.

### 3.5. Comparison of Automated Impaction Device and Manual Impaction

Baseline characteristics were generally similar between the AID and manual-impaction groups, although the stem-offset distribution differed between groups (*p* = 0.008) (Supplementary Table S2). AID utilization varied substantially by surgeon. Seven surgeons used both AID and manual impaction, contributing 442 cases to the within-surgeon analysis, including 384 AID cases and 58 manual cases (Table 6). In the surgeon fixed-effects model, AID use was not associated with operative time compared with manual impaction (adjusted difference, 0.18 min; 95% CI, −2.04 to 2.39; *p* = 0.8764). The results were similar after a case-mix adjustment (adjusted difference, 0.26 min; 95% CI, −1.83 to 2.36; *p* = 0.8053) (Table 6). No intraoperative complications occurred in either group. The single postoperative periprosthetic femoral fracture occurred in the AID group; no postoperative periprosthetic fractures occurred in the manual group. This comparison was reported descriptively without inferential testing because postoperative complications were uncommon. The unadjusted mean operative time was 21.0 ± 8.6 min for AID cases and 27.5 ± 8.4 min for manual cases, but this pooled difference was not interpreted as a device effect because of strong surgeon-level confounding.

## 4. Discussion

The principal findings of this multi-surgeon, multi-ASC series were low 90-day revision and complication rates following primary THA with a collared, triple-tapered cementless femoral stem, clinically meaningful short-term improvement in patient-reported outcomes, and no association between AID use and operative time after accounting for the surgeon. This study adds early clinical data for this specific collared triple-tapered stem in a same-day-discharge ambulatory pathway in which automated impaction was frequently used.

The low early revision and periprosthetic fracture rates observed in this cohort are consistent with prior reports of collared triple-tapered stem designs. However, the present study did not include a comparator stem or standardized radiographic analysis; therefore, the observed outcomes cannot be attributed directly to the stem geometry, collar, coating, or degree of osseointegration.

The observed 90-day periprosthetic fracture rate was low and within the range reported in contemporary studies of similar stem designs, although differences in follow-up duration and patient selection preclude a direct comparison. Dunleavy et al. reported a 90-day all-cause survivorship of 99.6% with a triple-taper stem, while a large multicenter registry study demonstrated a five-year Kaplan–Meier survivorship of 99.6% for stem revision with a very low periprosthetic femoral fracture rate of 0.06% [23,24]. Similarly, an American Joint Replacement Registry (AJRR) analysis of 8432 noncemented collared triple-taper stems implanted in patients older than 65 years between 2021 and 2024 demonstrated a two-year all-cause revision incidence of 1.32% and a periprosthetic femoral fracture (PPF) rate of 0.19% [25]. Salehi et al. further reported excellent short-term outcomes in 265 fully hydroxyapatite-coated, collared, triple-taper stems, with a two-year survivorship of 98.1%; the five revisions included two infections, two dislocations, and one acetabular periprosthetic fracture rather than femoral stem failure [26]. In the present cohort, all fractures occurred within the first 90 days, and no revisions were attributed to aseptic loosening. However, the short follow-up and absence of standardized radiographic assessment preclude conclusions regarding mechanical fixation, subsidence, or osseointegration.

Compared with prior generations of cementless femoral stems, these findings also appear favorable. Earlier tapered-wedge (“blade-type”) stems have demonstrated one-year all-cause survivorship rates of approximately 98.4%, with a fracture-free survivorship of 99.2% [27,28], while traditional proximal fit-and-fill stems have demonstrated a two-year Kaplan–Meier implant survivorship of 97.6% for all-cause revision [1]. Similarly, in a registry study by Hoskins et al. evaluating direct anterior THA, a traditional proximal fit-and-fill stem demonstrated 98.6% one-year survivorship compared with 98.0% for a tapered-wedge stem [29]. In another registry analysis evaluating cementless femoral stems, tapered-wedge stems demonstrated excellent 2-year survivorship, with cumulative percent revision (CPR) estimates ranging from 0.92 to 2.74% (97.3–99.1% survivorship), while traditional proximal fit-and-fill stems demonstrated similar outcomes, with 2-year CPR estimates ranging from 1.32 to 2.41% (97.6–98.7% survivorship) [30]. Collectively, prior studies provide useful context for the low early event rates observed here, but longer-term follow-up of the present cohort is required before conclusions regarding implant survival or fixation can be made.

The calcar collar is also a critical design feature, providing additional axial and rotational stability. In a series of 1888 THAs, collared stems demonstrated a periprosthetic fracture rate of 0.13% versus 1.42% for collarless stems (*p* = 0.002) [31]. Biomechanically, the collar transfers load to the resected femoral calcar, which fundamentally changes the strain distribution in the proximal femur, increasing axial compressive strains by 1147%, limiting subsidence and excessive seating in the early postoperative weeks [31,32,33,34]. However, collar contact, load transfer, subsidence, and implant migration were not measured in the present cohort; therefore, the current findings should not be interpreted as confirmation of these mechanisms.

The stem is composed of forged titanium alloy (Ti-6Al-4V) and incorporates a proximal hydroxyapatite coating intended to support biologic fixation [15,33,34,35,36,37]. Prior literature has associated hydroxyapatite-coated stems with lower rates of aseptic loosening and thigh pain [38]. However, osseointegration, subsidence, canal fill, radiolucent lines, and aseptic fixation were not directly evaluated in the present study because no standardized radiographic assessment was performed.

Our dislocation rate of 1.0% is comparable to prior nationwide data reporting a 1.4% dislocation-related readmission rate in the early postoperative period following primary THA [39,40,41]. Between 2012 and 2024, the American Joint Replacement Registry reports that the overall revision rates were between 0.9 and 1.4% [42]. Our 90-day all-cause revision rate of 0.4% is at the low end of this range.

Eighty-two percent of cases were performed with the AID. Prior studies have described potential ergonomic and procedural advantages of automated impaction, including reduced surgeon physical demand and more reproducible impaction [43,44,45,46]. In the present cohort, however, AID use was strongly associated with surgeon practice patterns. After accounting for surgeon, AID use was not associated with operative time. Accordingly, this study does not establish an operative-efficiency benefit attributable to the device. In addition, the absence of intraoperative complications in both groups should be interpreted descriptively because the study was underpowered to detect differences in uncommon safety events.

There was significant improvement in all PROMs, including VR-12 mental and physical components, HOOS-JR, and FJS. Patient satisfaction averaged 4.75 out of 5, with a high 95.8% of patients reporting “Satisfied” or “Very Satisfied.”

The principal limitation of this study is the short follow-up period. Although 90 days is sufficient to characterize early postoperative complications and revisions, it is insufficient to evaluate osseointegration, subsidence, aseptic loosening, or medium- and long-term implant survival. Although 522 of 525 hips had at least 90 days of effective follow-up, the overall follow-up period remained short and variable; therefore, these findings should be interpreted as early revision and complication rates rather than evidence of longer-term implant survivorship. The retrospective nature of this study necessitates reliance on the electronic medical record, which may underreport complications managed outside the facilities. This analysis was for the direct anterior approach at a same-day discharge ambulatory surgery center with a mean patient CCI of 2.46, which may affect generalizability to higher comorbidity burdens. The absence of a randomized AID-versus-manual comparator limits causal inference regarding automated impaction, and surgeon selection of impaction modality introduced substantial confounding. Although within-surgeon models were used, several surgeons contributed few cases with one of the two techniques. The low number of complications also left the study underpowered to detect differences in uncommon safety events between impaction methods. PROM responders differed modestly from nonresponders in BMI and selected baseline PROM scores, introducing the possibility of response bias (Supplementary Table S1). No standardized radiographic analysis was performed. Radiographs were obtained at variable postoperative time points and were reviewed clinically rather than according to a prespecified research protocol; therefore, conclusions regarding subsidence, component migration, canal fill, collar contact, radiolucent lines, biologic fixation, or osseointegration cannot be made. An effective follow-up was defined as the greater of documented clinical follow-up or 90 days for patients completing 12-week PROMs. Three hips had less than 90 days of effective follow-up but were retained because a complication occurred during the 90-day postoperative period. Further studies are warranted to assess whether the choice of surgical approach is a factor in this stem’s performance, as well as to identify mid- and long-term clinical outcomes, complications, and functional performance associated with this femoral stem.

## 5. Conclusions

In this retrospective ambulatory THA cohort, use of a collared, cementless, triple-tapered femoral stem was associated with low 90-day revision and complication rates and clinically meaningful short-term improvements in patient-reported outcomes. These findings are limited to the early postoperative period and do not establish osseointegration, aseptic fixation, or longer-term implant survival. AID use was not associated with operative time after accounting for surgeon; however, the low number of complications precluded a meaningful comparative safety analysis. A longer-term clinical and standardized radiographic follow-up is needed to evaluate component fixation, subsidence, osseointegration, and implant survival.

## Figures and Tables

**Table 1:** Demographics and surgical variables for all primary total hip arthroplasties (*n* = 525).

Demographic	Mean ± SD (Range)
Patient-level characteristics, *n* = 513 patients	
Age (Years)	66 ± 11 (23, 91)
Sex (% Men)	244 (48%)
BMI (kg/m^2^)	30 ± 6 (19, 47)
CCI	2.46 ± 1.37 (0, 9)
Hip-level characteristics, *n* = 525 hips	
Laterality (% Right)	288 (55%)
Preoperative diagnosis, *n* (%)	
Osteoarthritis	509 (97%)
Avascular Necrosis	12 (2.3%)
Legg-Calvé-Perthes disease	2 (0.4%)
Fracture	2 (0.4%)
Follow-up (Days)	110 ± 39 (29, 318)

Patient-level characteristics are reported among 513 unique patients. Hip-level characteristics are reported among 525 operated hips. Twelve patients underwent bilateral THA during the study period. SD, standard deviation; BMI, body mass index; kg/m^2^, kilograms/m^2^; CCI, Charlson Comorbidity Index.

**Table 2:** Surgical characteristics.

Surgical Characteristics	Total Hip Arthroplasties (*n* = 525)
Mean femoral stem size (range)	4.85 ± 2.02 (0.00, 12.00)
Femoral component offset; *n* (%)	
Standard	198 (38%)
High Offset	178 (34%)
Coxa Vara	149 (28%)
Head Size	
28	4 (0.8%)
32	43 (8.2%)
36	423 (81%)
40	55 (10%)
Wires/Cables	2 (0.4%)
Use of AID	432 (82%)

AID, Automated impaction device.

**Table 3:** Details of Reoperations.

CCI	Reason for Reoperation	Details	Days After Surgery?	Stem Size/Stem Offset/Head Size	AID Used?
3	Infection	DAIR with head, liner, and cup exchange. The stem was well fixed and saved.	34	4/CV/36	Yes
0	Concern for Fracture	Felt an immediate pop while going up the stairs (occult fracture) → one cable placed.	0	3/HO/36	Yes
3	Fracture	Felt pain overnight and reported to the emergency department (Vancouver B2) → stem revised and two cables placed.	15	2/CV/36	Yes

CCI, Charlson Comorbidity Index; DAIR, Debridement, Antibiotics, and Implant Retention; CV, Coxa Vara; HO, High Offset; AID, Automated Impaction Device.

**Table 4:** Details of medical and implant-related complications.

CCI	Complication	Details	Days After Surgery?	Stem Size/Stem Offset/Head Size
2	Dislocation	Closed reduced under sedation in ED and discharged the same day.	64	4/STD/32
3	Dislocation	Reduced in the ER and put in a knee immobilizer.	23	6/HO/36
4	Dislocation/Readmission	Closed reduction in the OR and admitted for further monitoring. Three days later, during hospitalization, the patient was treated for lower extremity cellulitis with Rocephin.	5	5/CV/36
3	Dislocation	Closed reduced under sedation in ED and discharged the same day.	21	3/CV/36
2	Dislocation	Reduced in the ER under sedation.	86	6/HO/36
4	Readmission	Presented to ED with dyspnea due to not taking anticoagulants. Admitted for 4 days and discharged once INR was at therapeutic levels.	60	5/STD/36
2	Readmission	Acute ischemic CVA, Afib with RVR. Admitted for 2 days.	27	7/CV/36
5	Readmission	Consulted their cardiologist for hypoxia and cardiac issues, who decided to admit the patient for 5 days. History of Ehlers–Danlos syndrome and POTS.	2	0/CV/32

CCI, Charlson Comorbidity Index; CV, coxa vara; HO, high offset; STD, standard; AID, automated impaction device; INR, International Normalized Ratio.

**Table 5:** Patient-reported outcome measures (PROMs): preoperative vs. 12 weeks postoperative.

PROM	Preoperative	Preoperative *n*	Postoperative (12 Weeks)	Postoperative *n* (12-Week)	Change: Preop → 12 Weeks (*p*-Value)	Paired *n*	MCID Achievement
VR-12 Mental (MCS)	49.9 ± 11.8	511	55.2 ± 8.8	486	+5.0 (<0.0001)	481	
VR-12 Physical (PCS)	31.0 ± 8.4	511	44.3 ± 8.9	486	+13.2 (<0.0001)	481	
HOOS-JR	51.3 ± 14.6	509	80.5 ± 14.7	489	+28.7 (<0.0001)	482	84.2%
FJS	12.1 ± 12.1	349	54.3 ± 27.9	439	+41.9 (<0.0001)	332	85.8%
Patient Satisfaction	N/A	N/A	4.75 ± 0.62	457	N/A	N/A	

Values are presented as means ± standard deviations unless otherwise indicated. Preoperative and 12-week values are based on available cases. Change scores and MCID achievement were calculated using paired complete cases only. No missing values were imputed. HOOS-JR MCID achievement was 406/482 (84.2%), and FJS MCID achievement was 285/332 (85.8%). Satisfaction data were available for 457 hips; 438/457 (95.8%) reported being satisfied or very satisfied. VR-12, Veterans RAND 12 Item Health Survey; MCS, Mental Component Score; PCS, Physical Component Score; HOOS-JR, Hip Disability and Osteoarthritis Outcome Score Joint Replacement; FJS, Forgotten Joint Score.

**Table 6:** Within-surgeon association between AID use and operative time.

Analysis	Adjusted Difference, AID—Manual, Min	95% CI	*p*-Value	Surgeons Included	AID Cases	Manual Cases
Surgeon fixed-effects model	0.18	−2.04 to 2.39	0.8764	7	384	58
Surgeon fixed effects plus case-mix adjustment	0.26	−1.83 to 2.36	0.8053	7	384	58

Negative estimates indicate a shorter operative time with AID. Analyses were restricted to surgeons who used both AID and manual impaction. AID, automated impaction device; CI, confidence interval.

## Data Availability

The data presented in this study are available on request from the corresponding author due to privacy restrictions.

## Supplementary Materials

The following supporting information can be downloaded at: https://www.mdpi.com/article/10.3390/jcm15166343/s1, Supplementary Table S1: Baseline Characteristics of PROM Responders and Nonresponders. Supplementary Table S2: AID versus manual baseline characteristics.

## Abbreviations

The following abbreviations are used in this manuscript:

AID	Automated Impaction Device
AJRR	American Joint Replacement Registry
ASC	Ambulatory Surgery Center
BMI	Body Mass Index
CCI	Charlson Comorbidity Index
DAIR	Debridement, Antibiotics, and Implant Retention
ED	Emergency Department
FJS	Forgotten Joint Score
HA	Hydroxyapatite
HOOS-JR	Hip Disability and Osteoarthritis Outcome Score for Joint Replacement
MCID	Minimal Clinically Important Difference
PPF	Periprosthetic Femur Fracture
POD	Postoperative Day
PROMs	Patient-Reported Outcome Measures
SDD	Same-Day Discharge
SD	Standard Deviation
THA	Total Hip Arthroplasty
Ti-6Al-4V	Titanium-6 Aluminum-4 Vanadium Alloy
VR-12	Veterans RAND 12-Item Health Survey
