# Supplementary material for: Early Performance of a Collared, Triple-Tapered Cementless Femoral Stem in Primary Total Hip Arthroplasty
Source: J Clin Med. 2026 Aug 17;15(16):6343. doi: 10.3390/jcm15166343 (PMC13513573; doi:10.3390/jcm15166343)
Supplement: Supplementary file 1 [file jcm-15-06343-s001.zip › jcm-4466128-supplementary.pdf]

Supplementary Table S1: Baseline Characteristics of PROM Responders and Nonresponders

Characteristic	No Paired PROM, N = 39	At Least One Paired PROM, N = 486	P value
Age, years	63 ± 12; 64 (57, 71)	66 ± 11; 67 (60, 74)	0.156
Sex, n (%)			0.286
Male	22 (56%)	231 (48%)	
Female	17 (44%)	255 (52%)	
BMI, kg/m²	32 ± 6; 30 (26, 38)	29 ± 6; 29 (26, 32)	**0.045**
CCI	2.26 ± 1.48; 2.00 (1.00, 3.00)	2.48 ± 1.36; 2.00 (2.00, 3.00)	0.221
Indication, n (%)			0.066
Osteoarthritis	36 (92%)	473 (97%)	
Avascular necrosis	2 (5.1%)	10 (2.1%)	
Femoral neck fracture	0	1 (0.2%)	
Legg-Calvé-Perthes disease	0	2 (0.4%)	
Trochanteric fracture	1 (2.6%)	0	
Impaction method, n (%)			0.692
Manual	6 (15%)	87 (18%)	
AID	33 (85%)	399 (82%)	
Preoperative VR-12 MCS	45 ± 11; 46 (36, 53)	50 ± 12; 51 (42, 60)	**0.018**
Preoperative VR-12 PCS	29 ± 8; 28 (23, 35)	31 ± 8; 30 (25, 37)	0.314
Preoperative HOOS-JR	44 ± 15; 47 (29, 53)	52 ± 14; 53 (43, 62)	**0.008**
Preoperative FJS	9 ± 11; 6 (0, 13)	12 ± 12; 8 (2, 19)	0.157

Values are presented as mean ± standard deviation; median (interquartile range), unless otherwise indicated. A PROM responder was defined as a hip with at least one paired preoperative and 12-week PROM. Continuous variables were compared using the Wilcoxon rank-sum test, and categorical variables were compared using the chi-square test or Fisher's exact test when expected cell counts were small. Missing values were not imputed. PROM, patient-reported outcome measure; BMI, body mass index; CCI, Charlson Comorbidity Index; AID, automated impaction device; VR-12, Veterans RAND 12-Item Health Survey; MCS, Mental Component Score; PCS, Physical Component Score; HOOS-JR, Hip Disability and Osteoarthritis Outcome Score for Joint Replacement; FJS, Forgotten Joint Score.

Supplementary Table S2: AID versus manual baseline characteristics

**Characteristic**	**Manual**<br>**N = 93**[1]	**AID**<br>**N = 432**[1]	**P value**
**Age (years)**	66 ± 11; 67 (61, 74)	66 ± 11; 67 (60, 73)	0.980
**Sex**			0.787
M	46 (49%)	207 (48%)	
F	47 (51%)	225 (52%)	
**BMI (kg/m²)**	30 ± 6; 28 (26, 32)	30 ± 6; 29 (26, 33)	**0.806**
**Charlson Comorbidity Index**	2.43 ± 1.27; 2.00 (2.00, 3.00)	2.47 ± 1.39; 2.00 (2.00, 3.00)	0.992
**Indication**			0.413
AVN	2 (2.2%)	10 (2.3%)	
FNF	1 (1.1%)	0 (0%)	
Legg-Calvé-Perthes disease	0 (0%)	2 (0.5%)	
OA	90 (97%)	419 (97%)	
Trochanteric fracture	0 (0%)	1 (0.2%)	
**Laterality**			0.821
R	52 (56%)	236 (55%)	
L	41 (44%)	196 (45%)	
**Stem size**	4.86 ± 2.15; 5.00 (3.00, 6.00)	4.84 ± 1.99; 5.00 (3.00, 6.00)	**0.937**
**Stem offset**			0.008
CV	15 (16%)	134 (31%)	
HO	41 (44%)	137 (32%)	
STD	37 (40%)	161 (37%)	
**Head size**			0.174
28	0 (0%)	4 (0.9%)	
32	12 (13%)	31 (7.2%)	
36	69 (74%)	354 (82%)	
40	12 (13%)	43 (10.0%)	

[1] Mean ± SD; Median (Q1, Q3); n (%). Values are presented as mean ± standard deviation; median (interquartile range), or n (%), as appropriate. Continuous variables were compared using the Wilcoxon rank-sum test, and categorical variables were compared using the chi-square test or Fisher's exact test when expected cell counts were small. AID, automated impaction device; BMI, body mass index; AVN, avascular necrosis; FNF, femoral neck fracture; OA, osteoarthritis; CV, coxa vara; HO, high offset; STD, standard.
